# Ligamentum teres reconstruction using autogenous semitendinosus tendon with toggle technique in rabbits

**DOI:** 10.7717/peerj.14777

**Published:** 2023-03-23

**Authors:** Rebeca Bastos Abibe, Sheila Canevese Rahal, Luciane dos Reis Mesquita, Danuta Doiche, Jeana Pereira da Silva, Maria Jaqueline Mamprim, Renata Haddad Pinho, Alexandre Battazza, Carlos Eduardo Fonseca Alves, W. Brian Saunders

**Affiliations:** 1 Department of Veterinary Surgery and Animal Reproduction, School of Veterinary Medicine and Animal Science, Universidade Estadual Paulista, Botucatu, São Paulo, Brazil; 2 Department of Veterinary Clinic, School of Veterinary Medicine and Animal Science, Universidade Estadual Paulista, Botucatu, São Paulo, Brazil; 3 Department of Small Animal Clinical Sciences, Texas A&M University, College Station, Texas, United States

**Keywords:** Hip joint, Luxation, Treatment, Surgery, Rabbit

## Abstract

**Background:**

Ligamentum teres (LT) has traditionally been considered a vestigial or redundant structure in humans; however, based on new studies and the evolution of hip arthroscopy, the LT injury has been viewed as a source of hip pain. Therefore, LT reconstruction can be beneficial in some cases. Rabbits have been frequently used as a model for cranial cruciate ligament reconstruction but few studies are available for ligamentum teres reconstruction.

**Objective:**

To evaluate the semitendinosus tendon to replace ligamentum teres with the toggle technique, using rabbits as an experimental model.

**Methods:**

Twenty-six female Norfolk rabbits with approximately 3 months of age were divided into two equal groups after excision of ligamentum teres (LT) from the right hip joint: G1—no reconstruction of LT and capsulorrhaphy; G2—double—bundle reconstruction of the LT using semitendinosus tendon autograft. In both groups, the LT was removed from the right hip joint. In G2 the autograft was harvested from the left hind limb of the same rabbit. The rabbits were evaluated clinically at different time intervals; before surgery (M1), 48 h (M2), 15 days (M3), 30 days (M4) and 90 days (M5) after surgery.

**Results:**

The rabbits supported their limbs on the ground in both the groups. As complications of the procedure, four hip joints showed subluxations in the radiographic evaluation of G1; three at M4 and one at M5. In G2; two luxations of hip joints at M3 and one subluxation at M4 were seen. On ultrasound, irregular articular surface was seen in 30.8% of the rabbits that had subluxation of hip joints. Gross evaluation identified tendon graft integrity in 76.92% of the rabbits. Histological analysis revealed graft adhesion to the bone in the early phase comprised of sharpey-like collagen fibers.

**Conclusion:**

The double-bundle reconstruction of the LT using autologous semitendinosus tendon associated with the toggle rod shows an early phase of tendon graft ligamentization at 90 days post-operatively in young rabbits, but biomechanical bias suffered by the tendon during gait must be considered.

## Introduction

In humans, the ligament of the femoral head or ligamentum teres (LT) has traditionally been considered a vestigial or redundant structure, with no biomechanical function or even vascularity, especially in the hip joint of adult individuals (Cerezal et al., 2010). Because of the uncertain function, the ligament has often been sacrificed in surgical procedures that require hip dislocation (Philippon, Pennock & Gaskill, 2012). Due to the advancement of studies on its structure and the evolution of hip arthroscopy, LT injury has been admitted as a source of hip pain in active patients, due to a large number of free nerve endings (Byrd & Jones, 2004).

Three groups of LT injuries have been determined based on arthroscopic analysis: complete rupture, usually associated with a traumatic episode or surgical procedure; partial rupture, seen in patients with a previous history of joint pain and degenerated ligament rupture associated with osteoarthritis (Gray & Villar, 1997). Some authors have suggested that patients with some degree of joint capsule laxity due to microtrauma induced by repetitive motions or with a complete or almost complete LT rupture due to traumatic or non-traumatic causes are among those who might get advantage from LT reconstruction (Amenabar & O’Donnell, 2012). The procedure has presented a good outcome in report cases in which autograft or allograft of the semitendinosus tendon (Amenabar & O’Donnell, 2012; Lindner et al., 2012), tibialis anterior allograft (White, Scoles & Herzog, 2018), or tibialis posterior tendon allograft (O’Donnell, Klaber & Takla, 2020) have been used for LT replacement.

On the other hand, an increase in displacement of the hip joint was observed after splitting LT in animal models (Cerezal et al., 2010; O’Donnell et al., 2014). Hip dislocation is a common traumatic injury in client-owned dogs and cats, representing up to 90% of all luxations in these species (DeCamp et al., 2016). In all cases, the LT is torn along with the tearing of a portion of the joint capsule (Tomlinson, 2014). The reduction and stabilization of hip dislocation have been treated in these cases by using closed or open techniques (Harasen, 2005; Moores, 2006; Tomlinson, 2014; DeCamp et al., 2016; Meeson & Strickland, 2021). Intra-articular or extra-articular surgical techniques and surgical procedures that alter the periarticular musculature and supporting structures have been described (Harasen, 2005). The toggle-rod stabilization is one of the intra-articular techniques that replace the LT with a suture (monofilament nylon or braided multifilament) and associated anchor (Harasen, 2005; Moores, 2006; Pratesi, Grierson & Moores, 2012; Kieves et al., 2014; Meeson & Strickland, 2021).

Rabbits have been frequently used as a model for cranial cruciate ligament reconstruction (Blickenstaff, Grana & Egle, 1997; Bachy et al., 2013; Giordano et al., 2015) but few studies are available for LT reconstruction, or even clinical case reports (Baek et al., 2018; Gallego & Villaluenga, 2019; Marinkovich et al., 2019). Thus, this study aimed to evaluate the semitendinosus tendon to replace LT with the toggle technique, using rabbits as an experimental model. The hypothesis was that the quadruped position and hopping gait of the rabbits may interfere with graft evolution.

## Materials and Methods

### Subjects

Twenty-six female Norfolk rabbits, approximately 3 months of age, weighing from 1.6 to 2.9 kg were used. The animals were obtained from the experimental animal center (EAC) from Sao Paulo State University (UNESP), Botucatu Campus. The EAC is associated with the department of animal science—rabbits and bioterium animals from the same institution.

The rabbits were randomly divided into two equal groups after a blood cell count (BCC) exam to evaluated previous health status: G1—no reconstruction of LT and capsulorrhaphy, G2—reconstruction of the LT using semitendinosus tendon autograft. Each rabbit was placed in an individual cage (60 × 60 × 60 cm) clear of the ground and with no restriction of movement, in an environment of approximately 27 °C temperature, controlled by exhaust fans and illumination for 12 h daily. The animals and the cages were identified with the number and the group of the rabbits. Environment was enriched with nipple-piped water, clay feed pot and banana tree leaf. Pinecone was given once a week. All the procedures of this study were executed in the Veterinary Hospital of UNESP.

### Anesthesia and surgical procedures

Rabbits were given ketamine (30 mg/kg, Dopalen; Lab. CEVA, Rockville, MD, USA) in combination with morphine (2 mg/kg, Dimorf; Cristalia, Itapira, Brazil) and midazolam (2 mg/kg, Dormire; Cristalia, Itapira, Brazil) intramuscularly. Approximately 10 min after, epidural anesthesia was performed with 2% lidocaine (Xylestesin; Cristalia, Itapira, Brazil). Then, rabbits were intubated for maintainance of anesthesia with isoflurane (Isoforine; Cristalia, Itapira, Brazil) by a certified anesthetist. Anesthesia monitorization comprised heart and respiratory rate, peripheral hemoglobin oxygen saturation, expired CO_2_ fraction (EtCO_2_) (multi-parameter monitor), non-invasive systolic arterial blood pressure (Doppler ultrasound), and rectal temperature (digital thermometer).

In G1, a craniolateral approach was used to approach the right hip joint. A partial tenotomy of the deep gluteal was done. The joint capsule was identified and incised along the ventral acetabular margin, taking care not to injure the cartilage. The LT was excised using a number 11 scalpel blade, and the femoral head was dislocated. After repositioning the femoral head in the acetabulum, the joint capsule was closed with a simple interrupted suture using polyglactin 910, in 5-0 size. A cruciate suture pattern was used to reattach the tendon of the deep gluteal muscle. The remaining closure was performed in a routine fashion.

In G2, a 5 cm longitudinal incision was made in the medial aspect of the left hind limb, from the medial condyle of the femur to the medial malleolus. The semitendinosus tendon was identified, carefully dissected from the surroundings, and harvested for its whole length (File S1). The fascia and soft tissue in the harvest site were closed in a simple continuous pattern using a 3-0 monofilament nylon suture. Skin apposition was done with a simple continuous intradermal pattern using the same suture material.

**Figure 1:**
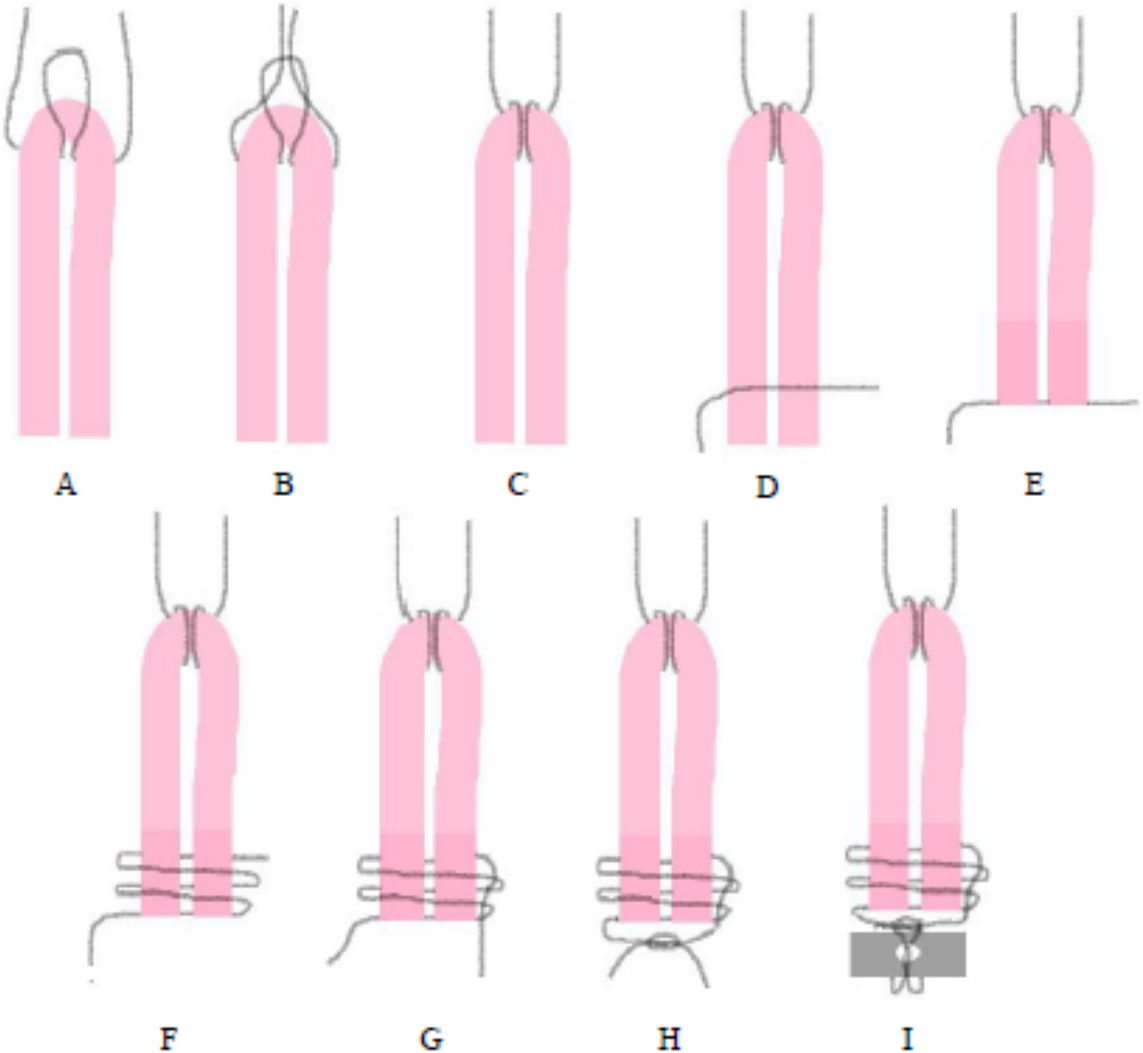
(A–I) Illustrative scheme. Preparation of a semitendinosus tendon graft for reconstruction of the ligamentum teres in rabbits using a double-bundle technique.

After harvesting, the tendon was folded into two equal parts (Fig. 1A). A loop with 2-0 nylon suture was applied at the folded end of the graft (Fig. 1B). The free ends of the nylon thread were passed through the loop and tensioned (Fig. 1C) in order to use posteriorly in the toggle rod. The two free ends of the graft were maintained together with other 2-0 nylon sutures (Figs. 1D and 1E). Two loops were made around the ends of the graft (Fig. 1F) and one of the free ends of the suture was passed inside the loops (Fig. 1G). Then, both ends of the suture were pulled and tied with the surgeon’s knot (Fig. 1H) in order to use posteriorly in the suture button. At the same time that the graft was prepared by an assistant, the right hip joint was approached as used in G1. The graft had a mean of 5.5 cm in length and 2 to 4 mm in width. A 3.2 mm hole was drilled through the acetabular fossa in the area of LT origin to accommodate the toggle rod and graft, and a bone tunnel was drilled (2.5 mm drill) starting at the level of the third trochanter, centering on the femoral neck and towards the fovea capitis with aid of C-shaped targeting guide. The graft was attached to the toggle rod (2.5 × 8 mm) that was inserted through the acetabular fossa. After the toggle rod was positioned to the medial aspect of the acetabulum, the graft was passed through the bone tunnel from the femoral head to the trochanter guided using a suture passer. Then, tension was placed on the tendon, which was tied to a button to the lateral aspect of the femur. The illustrative scheme of the preparation of semitendinosus tendon graft using a double-bundle technique is in Fig. 1 and File S2.

In both groups, on induction of anesthesia and for 5 consecutive days after surgery enrofloxacin (10 mg/kg sc q24h, Baytril; Bayer, Leverkusen, Germany), tramadol chlorhydrate (10 mg/kg q12h, Tramadol; John Martin, Chicago, IL, USA) and meloxicam (0.5 mg/kg q24, Maxicam; Ourofino, Cravinhos, Brazil) were administered subcutaneously.

### Clinical evaluations and imaging studies

The rabbits were evaluated before surgery (M1), 48 h (M2), 15 days (M3), 30 days (M4) and at 90 days (M5) after surgery by using visual gait analysis (presence or absence of lameness and/or hopping motion) (Miwa & Carrasco, 2019; McClure, 2020). For visual gait analysis, each animal was removed from the cage and allowed to walk freely in a quiet area with a concrete floor.

Except for M2, radiographic and ultrasound exams of the right hip joint were performed at the same time points. Radiographs were taken on extended ventrodorsal and right mediolateral projections. A digital radiography system (SR 8100; SIUI, Shantou, China) was used to obtain the images. The presence of hip dislocation or subluxation, toggle rod position, and signs of infection were assessed. Ultrasound (LOGIQ, GE Healthcare, Chicago, IL, USA) was used to assess joint capsule thickness, joint congruence, regularity of the articular surface, presence of osteophytes, and muscle organization. The images were obtained with an 8–13 MHz linear transducer. An experienced examiner evaluated the radiographic images and another one the ultrasound images.

### Gross and histological examination

At 90 days after surgery, all rabbits were euthanized. For euthanasia, the rabbits were submitted to dissociate anesthesia with xylazine hydrochloride (1 mg/kg, Xilazin; Syntec, London, England) and ketamine (50 mg/kg) administered in the same syringe intramuscularly. Subsequently, propofol (Propovan; Cristalia, Itapira, Brazil) was used intravenously until the respiratory arrest, followed by 19.1% potassium chloride (Cloreto de potassio; Samtec, New Albany, IN, USA) intravenously until the cardiac arrest. All the animals from both groups were euthanized to achieve the comparison from both treatments in gross and histological examination, following the relevant statistic number of samples.

In both groups the right hip joint was evaluated macroscopically for joint capsule thickness (mild, moderate, or intense); articular cartilage lesion of the femoral head (Grade 0—surface intact, Grade 1—superficial defect, Grade 2—deep defect, Grade 3—erosion with exposed subchondral bone) (Yoshioka et al., 1996); periarticular osteophytes and bone remodeling of the femoral head (mild or intense).

The hip joint of the G2 was fixed in a 10% buffered formalin solution for 48 h followed by nitric acid solution until decalcification (Bancroft & Gamble, 2008). Neutralization was accomplished by immersing decalcified bone in a 10% buffered formalin solution for 3 h. Specimens were cut into 4-μm sections and stained with hematoxylin and eosin. The organization and thickness of the conjunctive fibers from the joint capsule and tendon-bone interface were evaluated using light microscopy.

### Statistical analysis

The body mass values were analyzed using Student’s t-test for independent samples.

Statistical significance was set at *p* < 0.05. Data were analyzed using Minitab statistical software.

## Results

### Clinical evaluations and imaging studies

There was no statistically significant difference in body mass between G1 (2 ± 0.4 kg) and G2 (2.2 ± 0.4 kg) (File S3). One rabbit from G2 had a femur fracture at M3, and euthanasia was performed. Lameness was not recognized in both groups, but the rabbits from G2 showed more limitations for hopping motion until M3 compared to G1. Two rabbits from G2 with hip luxation showed the greatest limitation for hopping motion.

**Figure 2:**
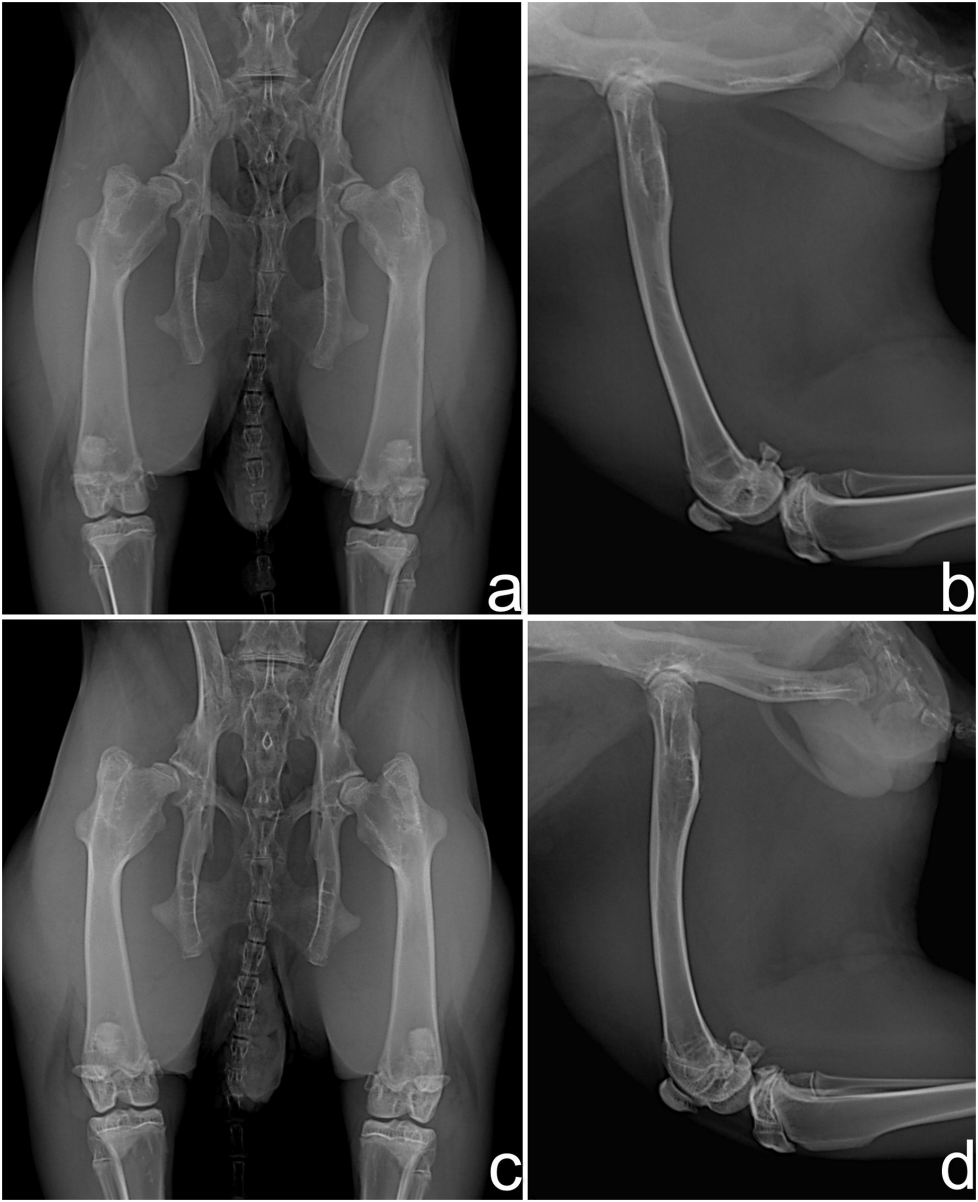
Ventrodorsal (A and C) and mediolateral (B and D) radiographic views at 90 days after surgery in rabbits of Group 1. Hip joints with no complication (A and B) and right hip joint with subluxation of the femoral head (C and D).

**Figure 3:**
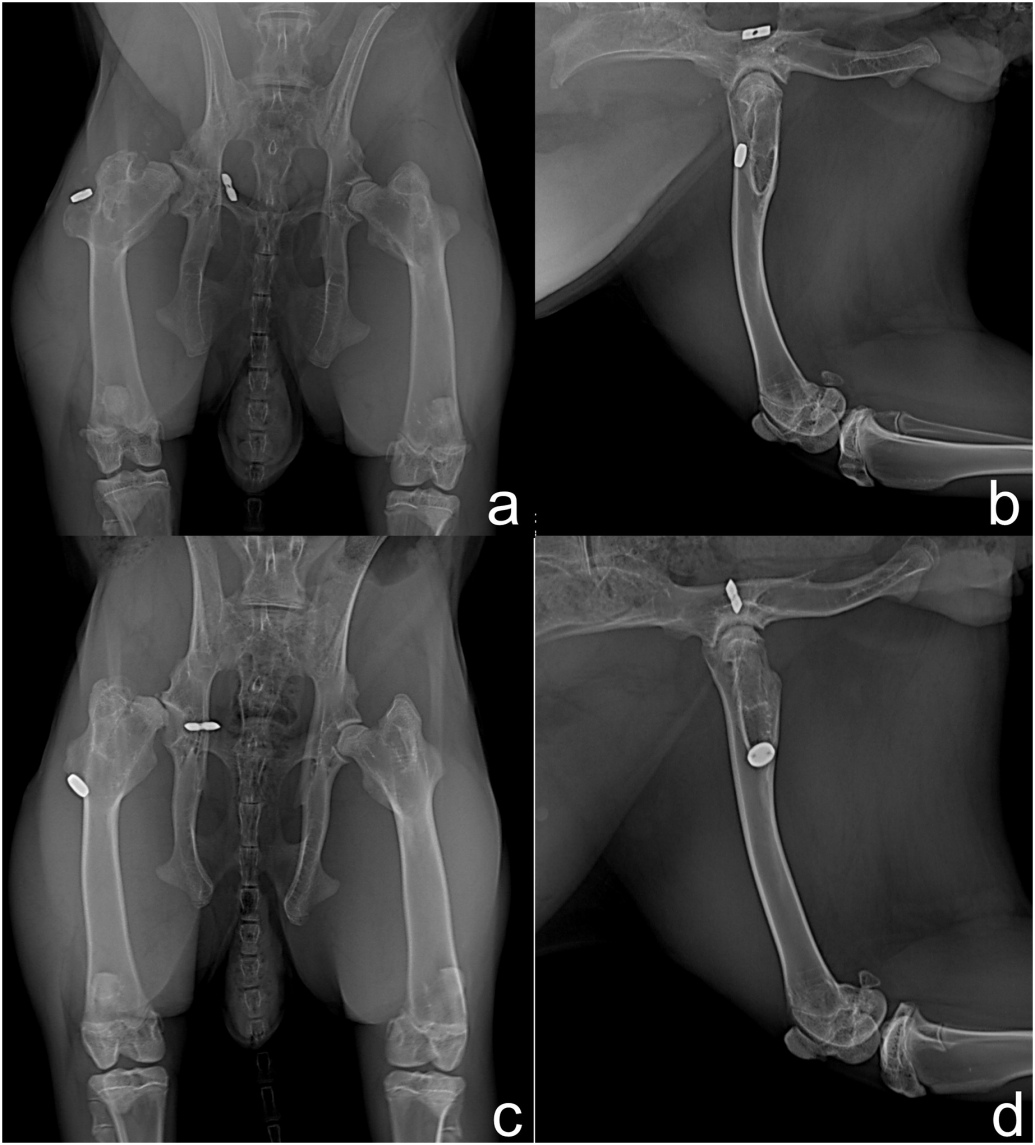
Ventrodorsal (A and C) and mediolateral (B and D) radiographic views at 90 days after surgery in rabbits of Group 2. Right hip joint with appropriate implant placement (A and B) and subluxation of the right hip joint due to toggle rod displacement (C and D).

In the radiographic evaluation of G1, nine rabbits showed no complications (Figs. 2A and 2B). However, hip joint subluxations in four rabbits were seen; in three rabbits at M4 and one rabbit showed subluxation at M5 (Figs. 2C and 2D). In G2, eight rabbits did not show any complication with the implant (Figs. 3A and 3B). Hip joint luxations in two rabbits at M3 and in one case subluxation at M4 were seen. As previously reported, one rabbit had a fracture of the right femur detected at M3. In addition, lytic lesions in the acetabulum, and periarticular tissue proliferation was observed in one rabbit at M4. In all cases of subluxation and luxation, the X-rays showed that the toggle rod had displaced toward the acetabular hole (Figs. 3C and 3D).

The sonographic findings in G1 revealed that the joint surface had no irregularities in 69.2% of the rabbits. The irregular articular surface was seen in 30.8% of the rabbits that had subluxated hips. LT was not seen in M3, M4, and M5. Disorganization of the orientation of the muscle fibers was seen at M3 that improved at M4 and M5. Three rabbits with hip subluxation at M4 and one rabbit with hip luxation at M5 had thickening of the joint capsule at M5. In G2, the articular surface had no irregularities on ultrasound at M1. Disorganization of the orientation of the muscle fibers and two hip luxations were seen at M3. One hip subluxation with severe signs of osteoarthritis was observed at M4. Thickening of the joint capsule was detected in the two hip luxations, and in the one hip subluxation. Improved muscle fiber organization was seen at M5. The two hip luxations and the hip with osteoarthritis had irregular articular surfaces.

### Gross and histological examination

The gross evaluation of G1 showed mild capsular thickness and mild remodeling of the femoral head in 30% and no alteration in 70% of rabbits. Articular cartilage lesions were classified as 23.1% of rabbits in Grade 0, 15.4% in Grade 1, 30.8% in Grade 2, and 30.8% in Grade 3, which also presented joint subluxation.

**Figure 4:**
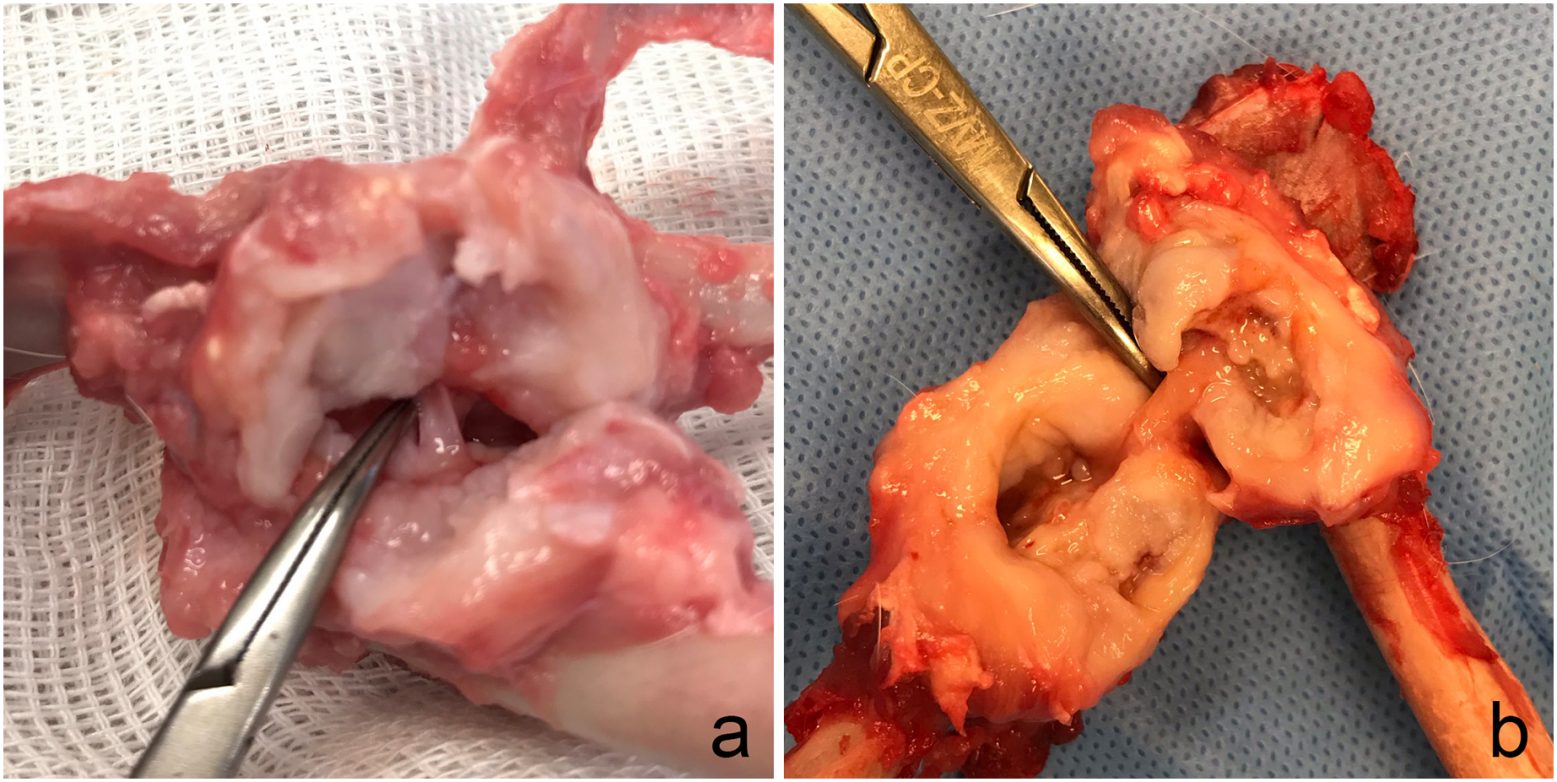
Gross evaluation. Note the integrity of the semitendinosus tendon graft used to replace ligamentum teres in two rabbits (A and B) of Group 2.

The gross evaluation of G2 showed 53.8% mild, 46.2% moderate, and 15.4% intense capsular thickness. In addition, articular cartilage lesions were classified 30.8% rabbits in Grade 1, 38.4% Grade 2, and 30.8% Grade 3. Two rabbits with Grade 3 (15.4%) had hip luxation due to graft failure (resorption or toggle rod displacement). The integrity of the tendon graft (Fig. 4) was verified in 76.92% of the rabbits including the rabbit euthanatized 15 days after surgery (M3) due to femur fracture. Two rabbits (15.4%) showed rupture of the tendon graft in its proximal anchorage (acetabulum) associated with hip luxation. One rabbit (7.7%) showed partial rupture of the intra-articular tendon graft.

**Figure 5:**
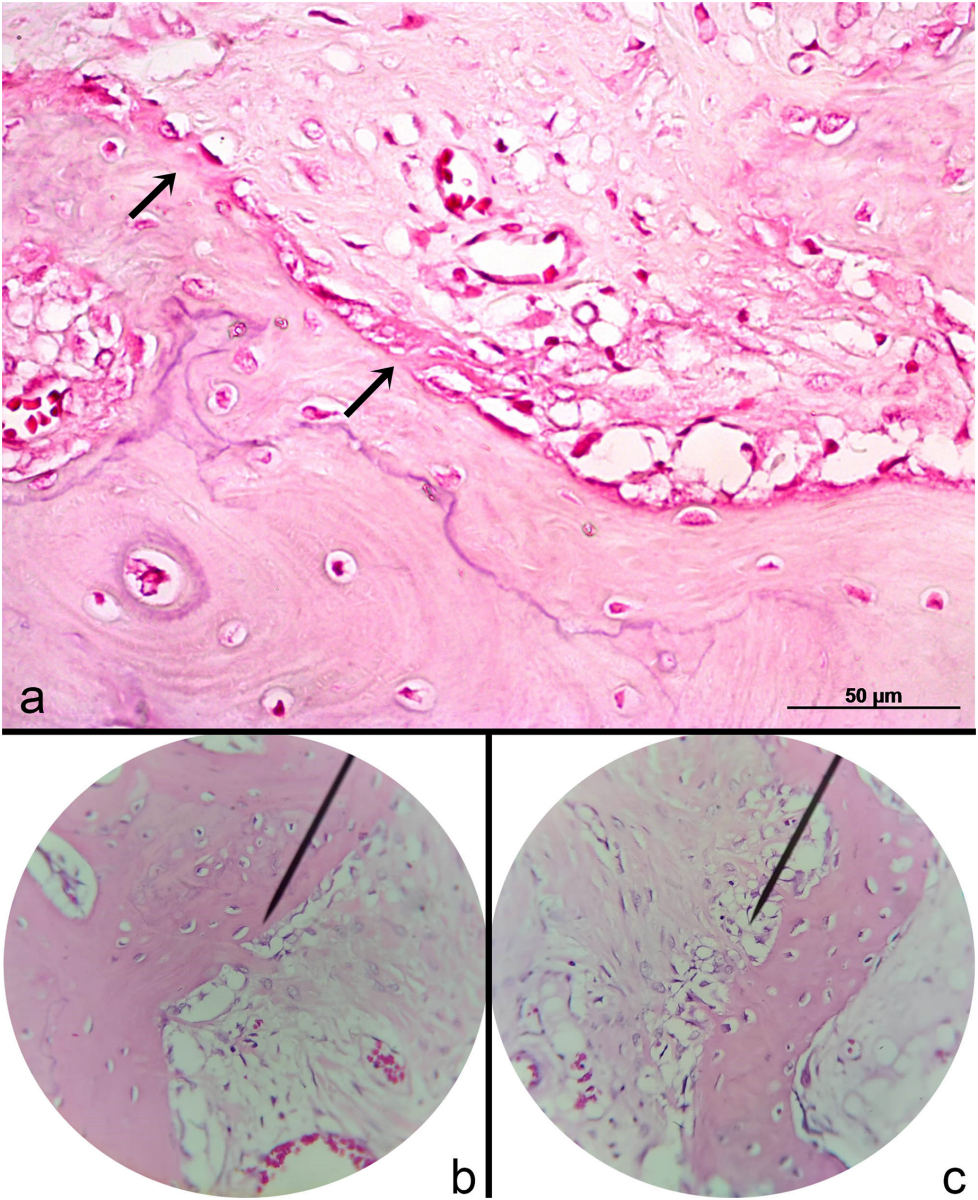
Photomicrographs of the periosteal collagen fibers (Sharpey-like fibers) at the tendon-bone interface. (A) The early stage of the fibers (arrows) and viable blood vessels can be visualized HE 400X; (B) advanced stage. HE 200X; (C) mild stage spread along the bone-graft interface. HE 200X.

Histological analysis revealed characteristics of the early phase of tendon graft ligamentization with widely diffuse and homogeneous multifocal areas of collagen fibers, consistent with sharpey fibers at the tendon-bone interface (Fig. 5). The fibers were in the early phase to bind the periosteum accompanying the presence of organized connective tissue. Blood vessels were seen in the graft concomitant to 1–3 chondrocyte layers bordering the bone graft. G2 had a higher rate of fissures and irregularity in the cartilage surface compared to G1. Joint capsule thickness was observed. However, joint capsule fibers had preserved architecture.

## Discussion

The present study showed that the semitendinosus tendon graft may be used to replace LT using the toggle-rod technique; however, some complications were encountered. The LT was chosen due to the ease of harvest, length, and minimal donor site morbidity. The grafts of LT, semimembranosus tendon and patellar ligaments are the most commonly used in rabbits, especially for replacing the cranial cruciate ligament (Bi et al., 2015; Giordano et al., 2015). LT is most recommended in immature skeletons due to the risk of physeal closure and bone deformity with a patellar tendon graft (Giordano et al., 2015). This premise was also considered in the current study due to the rabbit’s age. The gait of the rabbits was not compromised by graft harvesting because the common calcaneal tendon is composed of the calcaneal tendon of the semitendinosus muscle, the triceps surae, and the superficial digital flexor tendon (Skalec, Janeczek & Czerski, 2019), the LT excision was probably compensated for by the other musculotendinous groups.

The tendon allograft was prepared using a double-bundle technique, similar to that described for LT replacement in human patients (Amenabar & O’Donnell, 2012; Lindner et al., 2012; O’Donnell, Klaber & Takla, 2020). However, despite the tendon of the semitendinosus muscle being considered thin but strong (Skalec, Janeczek & Czerski, 2019), the small diameter of the tendon graft in rabbits made it hard to take the sutures through the tendon, and suture loops were used.

The visual gait analysis did not help to detect complications, since rabbits presenting hip luxation (G2) did not show lameness. On the other hand, non-weight bearing lameness was observed in a pet rabbit with hip luxation that was solved after using a toggle pin and nylon suture (Marinkovich et al., 2019).

The ultrasound appearance of muscle showed more organized tissue structure in both groups at M5, suggesting a recovery process despite the type of surgical procedure. On the other hand, radiographs and ultrasonography of hip joints revealed a higher number of complications in G2 compared to G1. Although the hip joints in G1 were stabilized with capsulorrhaphy, the joint exposure was lesser, justifying only the occurrence of subluxations (*n* = 4). Hip luxations (*n* = 2) and subluxation (*n* = 1) found in G2 were due to toggle-rod displacement. Studies in dogs and cats using different types of toggle rods and suture material to replace the LT have also reported complications, including luxation, premature rupture of the suture, toggle rod pullout or breakage, and infection (Kieves et al., 2014; Trostel & Fox, 2020; Mathews & Barnhart, 2021). Luxation after the toggle technique has ranged from 3% to 14.8% in dogs and cats (Demko et al., 2006; Pratesi, Grierson & Moores, 2012; Mathews & Barnhart, 2021). Probably the use of a larger size toggle rod could prevent pullout in G2.

The rabbit hopping motion using the vigorous hindlimbs (McClure, 2020) transfers the load to the implant and may contribute to complication risk. Activity restriction or placement of limbs in non-weight bearing slings have been used to minimize postoperative complications in small animals with traumatic hip luxation treatment using the toggle technique (Moores, 2006; Kieves et al., 2014; Trostel & Fox, 2020; Mathews & Barnhart, 2021). In human patients, limited weight-bearing support is also indicated for some weeks after the surgical procedure (Lindner et al., 2012; Philippon, Pennock & Gaskill, 2012). Some of these interventions are difficult to use in rabbits, representing a limitation of this experimental model. Despite this, the integrity of the tendon graft was verified in 76.92% of the rabbits, suggesting biomechanical strength. The two cases of tendon rupture were in the toggle rod anchorage, suggesting this tendon end need to be reinforced.

The macroscopic analysis confirmed that two rabbits showed toggle rod displacement with partial or total graft failure. Since the graft cannot be identified in total failure, the hypothesis was that of a graft resorption process. Graft resorption was also seen in a human patient with TL rupture treated with semitendinosus tendon, which was attributed to the impact on the graft (Amenabar & O’Donnell, 2012). Factors such as infection, impact, and excessive tension in the tendon graft cause inadequate vascularization and favor that incorporation does not occur (George, Dunn & Spindler, 2006). In the present study, the most probable causes were the impact on the tendon and tension.

Excluding the hip subluxations and luxations in both groups, the imaging exams and gross evaluation found more cartilage lesions in G2 than G1. Dogs treated with open reduction and toggle pin/rod stabilization with wire had 5.5% of osteoarthritis (Mathews & Barnhart, 2021). Arthroscopic reconstruction of the LT has been performed in human patients (Philippon, Pennock & Gaskill, 2012; Lindner et al., 2012; O’Donnell, Klaber & Takla, 2020), which minimizes trauma compared to traditional open surgery. Furthermore, the capsular incision to expose the femoral head and acetabulum in open procedures contribute to a reduction in local stability. Joint instability has been associated with the development of osteoarthritis (Blalock et al., 2015).

Histologically, the interface between the tendon and the bone (femoral head, acetabulum) had periosteal collagen fibers (sharpey’s fibers). In one study of rabbits with intraarticular semitendinosus autograft positioned through tibial and femoral holes observed the blending of the graft to the fibrous connective tissue over time that adhered to bony trabeculae (Blickenstaff, Grana & Egle, 1997). Healing of tendon-bone interface aiming at an effective ligament reconstruction can be slowed because of the blood supply deficit due to vascular and bone loss in the drilling site (Bi et al., 2015). In the present study, a partial adhesion of collagen fibers to the periosteum was found 90 days after surgery, suggesting an effect of surgical injury.

The joint capsule fibers had preserved architecture on histology. A biomechanical study did not detect differences between hip capsulorrhaphy in block suture whipstitch technique with polyglycolic acid or titanium anchors in rabbits (Garcia Filho et al., 2012). Capsular incision and sutures contribute to capsular thickening that was more intense with hip dislocation, probably in an attempt to improve joint stabilization.

The present study has some limitations. The surgical approach to the hip joint required a longer incision for optimal exposure of the femoral head that contributed to postoperative hip luxation; however, an arthroscopic surgical procedure in the rabbit hip joint is technically difficult and semitendinosus graft length allows only the double-bundle technique. Thus, reconstruction needs to be tested with other tendon grafts.

## Conclusions

The double-bundle reconstruction of the LT using autologous semitendinosus tendon associated with toggle-rod shows an early phase of tendon graft at 90 days post-operatively in young rabbits. The potential for complications, such as femur fracture and graft/toggle failure, does exist and should be the focus of future studies.

## Supplemental Information

10.7717/peerj.14777/supp-1 Supplemental Information 1 Exposition of the semitendinosus tendon for harvesting, and the length of semitendinous tendon graft after harvesting.

10.7717/peerj.14777/supp-2 Supplemental Information 2 Preparation of semitendinosus tendon graft using a double-bundle technique.

10.7717/peerj.14777/supp-3 Supplemental Information 3 Body mass values. Values in Group 1 and Group 2.

## Additional Information and Declarations

### Data Availability

The following information was supplied regarding data availability: The data is available at Respositório Institucional UNESP: Round ligament reconstruction using tendon autograft, http://hdl.handle.net/11449/215401.

## References

[ref-1] Amenabar T, O’Donnell J (2012). Arthroscopic ligamentum teres reconstruction using semitendinosus tendon: surgical technique and an unusual outcome. Arthroscopy Techniques.

[ref-2] Bachy M, Sherifi I, Zadegan F, Petrover D, Petite H, Hannouche D (2013). Anterior cruciate ligament surgery in the rabbit. Journal of Orthopaedic Surgery and Research.

[ref-3] Baek JH, Chun YS, Rhyu KH, Yoon WK, Cho YJ (2018). Effect of ligamentum teres tear on the development of joint instability and articular cartilage damage: an *in vivo* rabbit study. Anatomical Science International.

[ref-4] Bancroft JD, Gamble M (2008). Theory and practice of histological techniques.

[ref-5] Bi F, Shi Z, Liu A, Guo P, Yan S (2015). Anterior cruciate ligament reconstruction in a rabbit model using silk-collagen scaffold and comparison with autograft. PLOS ONE.

[ref-6] Blalock D, Miller A, Tilley M, Wang J (2015). Joint instability and osteoarthritis. Clinical Medicine Insights: Arthritis and Musculoskeletal Disorders.

[ref-7] Blickenstaff KR, Grana WA, Egle D (1997). Analysis of a semitendinosus autograft in a rabbit model. American Journal of Sports Medicine.

[ref-8] Byrd JW, Jones KS (2004). Traumatic rupture of the ligamentum teres as a source of hip pain. Arthroscopy.

[ref-9] Cerezal L, Kassarjian A, Canga A, Dobado MC, Montero JA, Llopis E, Rolón A, Pérez-Carro L (2010). Anatomy, biomechanics, imaging, and management of ligamentum teres injuries. Radiographics.

[ref-10] DeCamp CE, Johnston SA, Déjardin LM, Schaefer SL, DeCamp CE, Johnston SA, Déjardin LM, Schaefer SL (2016). The hip joint. Handbook of Small Animal Orthopedics and Fracture Repair.

[ref-11] Demko JL, Sidaway BK, Thieman KM, Fox DB, Boyle CR, McLaughlin RM (2006). Toggle rod stabilization for treatment of hip joint luxation in dogs: 62 cases (2000–2005). Journal of the American Veterinary Medical Association.

[ref-12] Gallego M, Villaluenga JE (2019). Coxofemoral luxation in pet rabbits: nine cases. The Journal of Small Animal Practice.

[ref-13] Garcia Filho FC, Guarniero R, Godoy Júnior RM, Pereira CAM, Matos MA, Garcia LC (2012). Simple suture and anchor in rabbit hips. Acta Ortopedica Brasileira.

[ref-14] George MS, Dunn WR, Spindler KP (2006). Current concepts review: revision anterior cruciate ligament reconstruction. The American Journal of Sports Medicine.

[ref-15] Giordano M, Falciglia F, Poggiaroni A, Aulisa AG, Savignoni P, Guzzanti V (2015). Histological changes of semitendinosus autograft after anterior cruciate ligament reconstruction in an immature rabbit model. Journal of Experimental Orthopaedics.

[ref-16] Gray AJ, Villar RN (1997). The ligamentum teres of the hip: an arthroscopic classification of its pathology. Arthroscopy.

[ref-17] Harasen G (2005). Coxofemoral luxations—part 2: surgical options. Canadian Veterinary Journal.

[ref-18] Kieves NR, Lotsikas PJ, Schulz KS, Canapp SO (2014). Hip toggle stabilization using the TightRope® system in 17 dogs: technique and long-term outcome. Veterinary Surgery.

[ref-19] Lindner D, Sharp KG, Trenga AP, Stone J, Stake CE, Domb BG (2012). Arthroscopic ligamentum teres reconstruction. Arthroscopy Techniques.

[ref-20] Marinkovich M, Guzman DS-M, Hawkins MG, Gleeson M, Chou P-Y (2019). Open reduction and stabilization of a luxated coxofemoral joint in a domestic rabbit (*Oryctolagus cuniculus*) using a toggle-pin fixation. Journal of Exotic Pet Medicine.

[ref-21] Mathews ME, Barnhart MD (2021). Risk factors for reluxation after toggle rod stabilization for treatment of coxofemoral luxation in 128 dogs. Veterinary Surgery.

[ref-22] McClure D (2020). Description and physical characteristics of rabbits. Merck veterinary manual.

[ref-23] Meeson RL, Strickland R (2021). Traumatic joint luxations in cats: reduce, repair, replace, remove. Journal of Feline Medicine and Surgery.

[ref-24] Miwa Y, Carrasco DC (2019). Exotic mammal orthopedics. Veterinary Clinics of North America: Exotic Animal Practice.

[ref-25] Moores A (2006). Decision making in the management of hip luxations in dogs and cats. In Practice.

[ref-26] O’Donnell J, Klaber I, Takla A (2020). Ligamentum teres reconstruction: indications, technique and minimum 1-year results in nine patients. Journal of Hip Preservation Surgery.

[ref-27] O’Donnell JM, Pritchard M, Salas AP, Singh PJ (2014). The ligamentum teres-its increasing importance. Journal of Hip Preservation Surgery.

[ref-28] Philippon MJ, Pennock A, Gaskill TR (2012). Arthroscopic reconstruction of the ligamentum teres: technique and early outcomes. The Journal of Bone and Joint Surgery. British Volume.

[ref-29] Pratesi A, Grierson J, Moores AP (2012). Toggle rod stabilisation of coxofemoral luxation in 14 cats. The Journal of Small Animal Practice.

[ref-30] Skalec A, Janeczek M, Czerski A (2019). Anatomy and histology of the rabbit common calcanean tendon. Anatomia, Histologia, Embryologia.

[ref-31] Tomlinson JL, Bojrab MJ, Waldron DR, Toombs JP (2014). Treatment of coxofemoral luxations. Current Techniques in Small Animal Surgery.

[ref-32] Trostel CT, Fox DB (2020). Coxofemoral joint luxation in dogs treated with toggle rod stabilization: a multi-institutional retrospective review with client survey. Journal of the American Animal Hospital Association.

[ref-33] White BJ, Scoles AM, Herzog MM (2018). Simultaneous acetabular labrum and ligamentum teres reconstruction: a case report. Journal of Hip Preservation Surgery.

[ref-34] Yoshioka M, Coutts RD, Amiel D, Hacker SA (1996). Characterization of a model of osteoarthritis in the rabbit knee. Osteoarthritis and Cartilage.

