# Supplementary material for: Ligamentum teres reconstruction using autogenous semitendinosus tendon with toggle technique in rabbits
Source: PeerJ. 2023 Mar 23;11:e14777. doi: 10.7717/peerj.14777 (PMC10040178; doi:10.7717/peerj.14777)
Supplement: Supplemental Information 1 [file peerj-11-14777-s001.pdf]

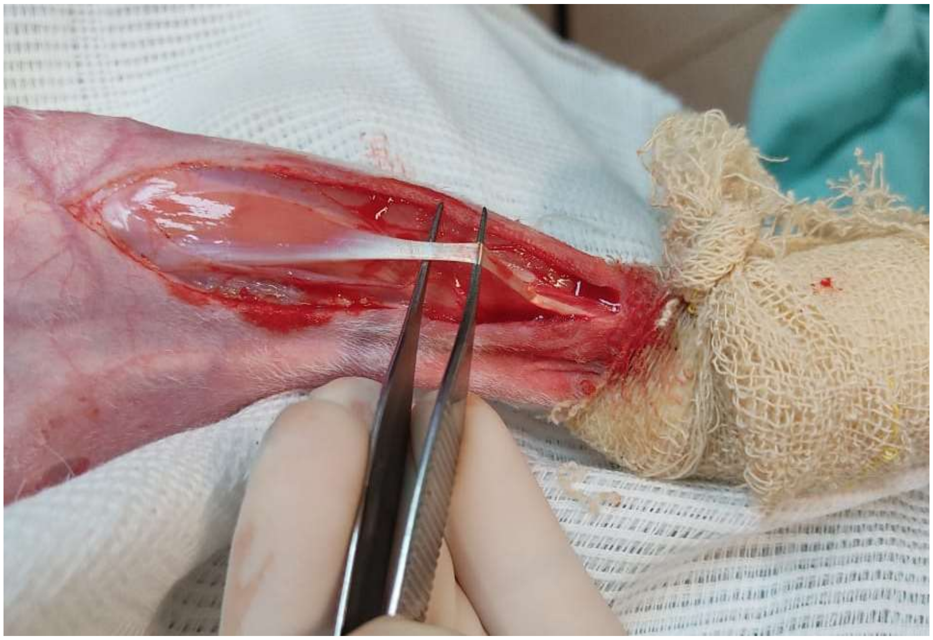

Exposition of the semitendinosus tendon for harvesting

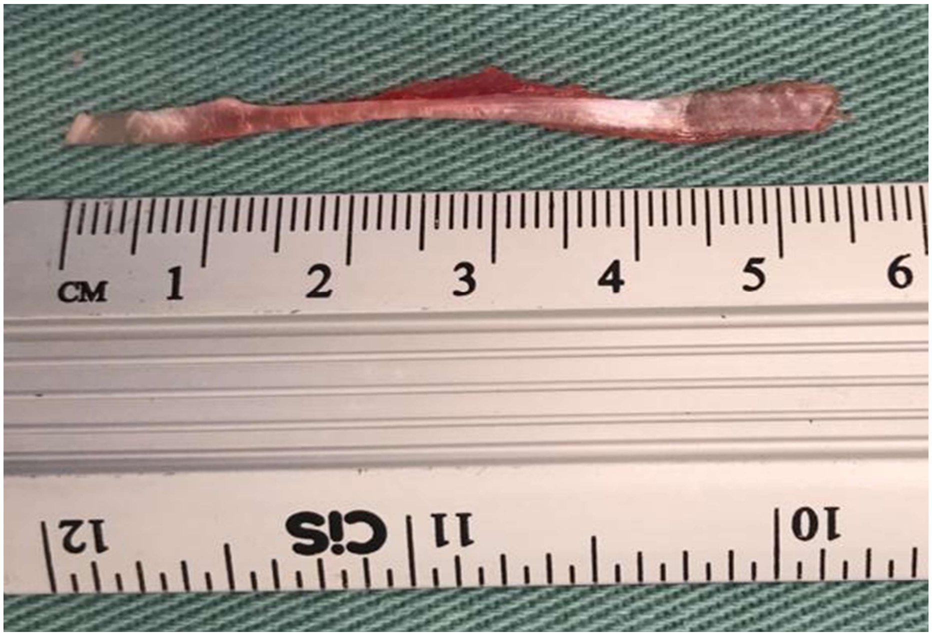

The length of semitendinous tendon graft after harvesting.
