# Supplementary figures and images for: Ligamentum teres reconstruction using autogenous semitendinosus tendon with toggle technique in rabbits
Source: PeerJ. 2023 Mar 23;11:e14777. doi: 10.7717/peerj.14777 (PMC10040178; doi:10.7717/peerj.14777)

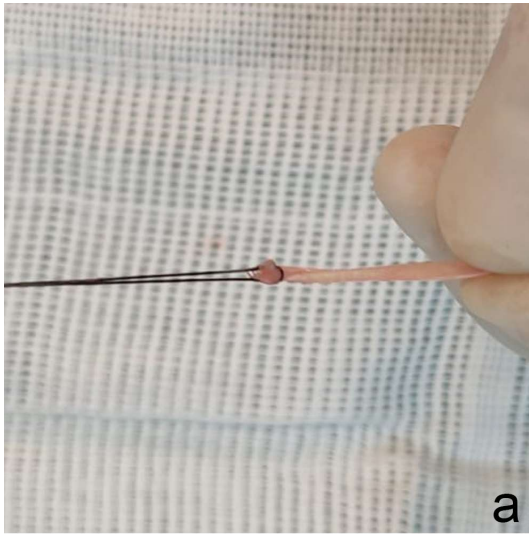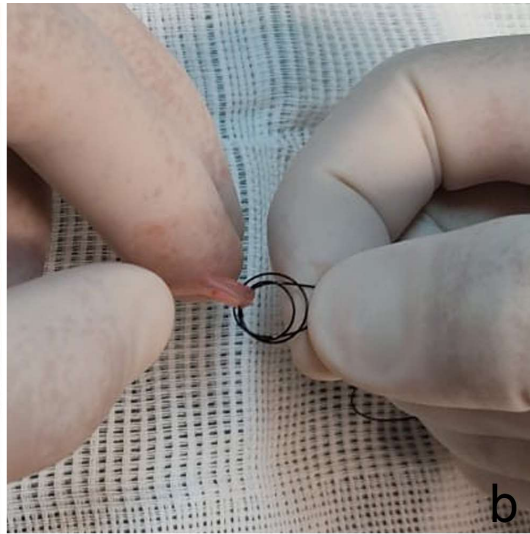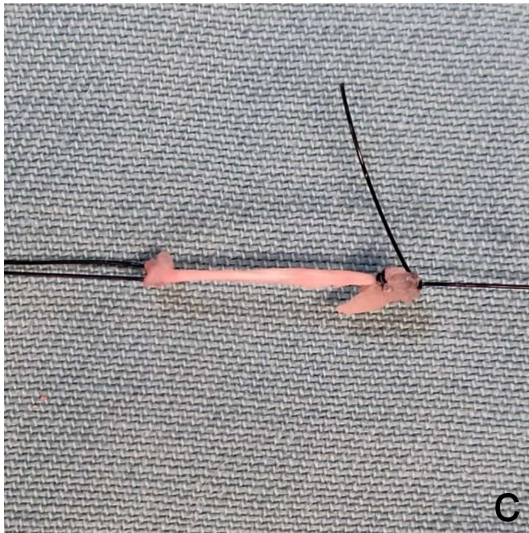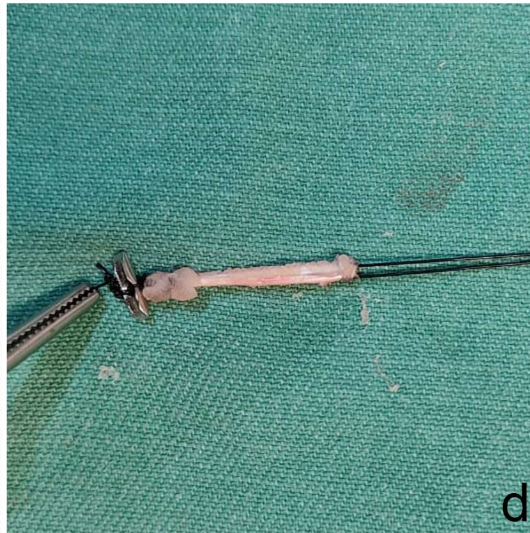

Preparation of semitendinosus tendon graft using a double-bundle technique.

Supplement: Supplemental Information 2 [file peerj-11-14777-s002.pdf]
